# Erosive tooth wear: what are UK undergraduates being taught?

**DOI:** 10.1038/s41415-026-9530-0

**Published:** 2026-05-08

**Authors:** Rupert Austin, Jonathan Creeth, Steve Mason, David Bartlett, Nigel Pitts

**Affiliations:** 421720678472765967984 Centre for Oral, Clinical & Translational Sciences, King´s College London Faculty of Dentistry, Oral & Craniofacial Sciences, United Kingdom; 420815859888136059630 Haleon, Weybridge, United Kingdom; 940246970968017402484 Retired, formerly Haleon, Weybridge, United Kingdom

## Abstract

**Introduction** The evidence-base for erosive tooth wear (ETW) is developing with greater awareness of both the impact of the condition and thus the importance of risk assessment using screening tools such as the basic erosive wear examination (BEWE).

**Aim** To survey what is currently being taught in UK dental schools regarding ETW.

**Materials and methods** A cross-sectional survey of selected ETW educators from UK dental schools was administered online.

**Results** The response rate was 16 (100% of UK dental schools). Fifty-six percent of UK dental schools report using a subject specific structure (akin to caries or periodontal disease) for teaching ETW. Ninety percent reported using ETW-specific recommendations from international curricula guidance (Organisation of Caries Research and the Association of Dental Education in Europe 2010 core cariology curriculum guidance). Ninety-two percent often/always taught BEWE screening and 75% felt that their dental learners were likely/very likely to be using BEWE routinely.

**Discussion** UK undergraduate educators teaching ETW report high levels of engagement with implementation of the international guidance and ETW screening/risk assessment.

**Conclusion** UK undergraduate dental teaching has almost universally adopted ETW screening and risk assessment using the BEWE. International guidance on ETW education is changing UK teaching practice with a greater emphasis on a research-informed educational approach.

## Key points

- The basic erosive tooth wear is taught almost universally in UK dental schools.
- Erosive tooth wear education in the UK is informed by international educational guidance.
- It is important to maintain academic expertise in the field of erosive tooth wear in UK dental schools.

## Introduction

Erosive tooth wear (ETW) is the third most observed oral condition impacting oral health in the UK, after caries and periodontal disease.^1^ ETW is caused by a combination of acid erosion, from extrinsic (dietary) or intrinsic (gastric) acid sources, that demineralise and hence soften the outer surfaces of tooth enamel and dentine, and physical challenges, namely tooth-tooth attrition or abrasion from toothbrushing and other causes that remove softened mineral.^2^ Enamel wear is thought to develop slowly (over years), to be irreversible and to progress when sufficient acidic and physical challenges exist.^3^ Diet and lifestyle factors are key drivers of the condition so careful assessment, risk management and patient education have a critical role to play in preventing progression of the condition.^4^^,^^5^

**Fig. 1:**
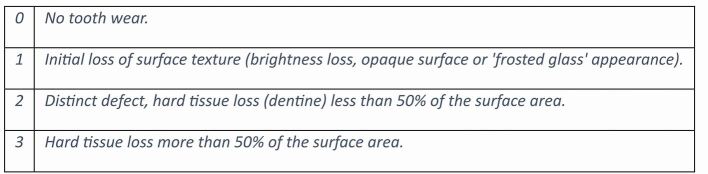
The basic erosive wear examination is a simple scoring system used to assess the severity of tooth wear, with scores ranging from 0 (no wear) to 3 (hard tissue loss affecting more than 50% of the surface). The most severely affected surface in each sextant is recorded. The cumulative score guides the management of the condition for the practitioner

**Fig. 2:**
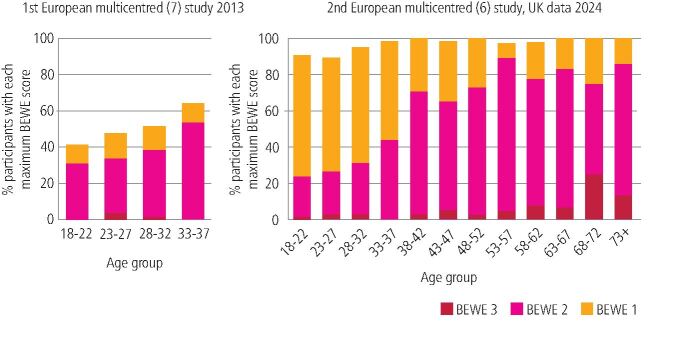
Prevalence data comparing the changes in basic erosive wear examination scores for UK adults between 2013 and 2024 (reproduced from Professor Nicola West with permission)

The basic erosive wear examination (BEWE) was introduced in 2008 as a screening and risk assessment tool for ETW^6^ and is now included in most practice management software, clinically validated and widely adopted in UK general dental practice.^7^ The BEWE is also used to provide prevalence data showing significant increases in the levels of ETW in the UK and Europe over the last decade. Prevalence data from UK 18–35-year-olds (2024), revealed that levels of ETW have increased from 29.4% to 47.7% over the last decade (i.e., distinct tooth wear lesions maximum BEWE score 2 or 3) (Fig. 1).^8^ Thus in 2024, 97.6% of UK adults had at least one surface with evidence of initial surface loss (BEWE 1) (Fig. 2). While this is a dramatic change, it is not clear if UK undergraduate teaching has kept pace with these data. Education needs to be research-informed, to ensure that learners are aware of global impact of the condition and how best to screen, risk assess and discuss preventive advice for their individual patients.

Educational guidelines to guide curricula development based on expert consensus support teaching of the term ETW, which promotes a concept of tooth wear as ‘the cumulative surface loss of mineralised tooth substance due to physical or chemo-physical processes (dental erosion, attrition, abrasion)' and ETW being ‘tooth wear with dental erosion as the primary etiological factor'.^2^ This is supported by the European Organisation for Caries Research (ORCA)^9^ and the Association for Dental Education in Europe ‘cariology core curriculum' (CCC) educational curricula guidance.^10^ This educational guidance includes specific recommendations on teaching prevention, risk assessment and diagnosis of ETW; however, it is not known to what extent this has been used for designing and delivering curricula in the UK.^11^

Thus, there is need to understand to what extent UK undergraduate teaching of ETW has kept pace with these developments, specifically which method for screening and risk assessment is being taught and what terminology educators are using when discussing the condition. Therefore, the aim of this study was to conduct a cross-sectional survey of UK dental schools to identify what is currently being taught to undergraduates in the field of ETW and to identify dental schools' priorities for the education of this important condition.

## Materials and methods

A cross-sectional survey was conducted, using a previously validated questionnaire, modified to focus specifically on the educational aspects of ETW. Ethical approval for the questionnaire was granted by King's College London (registration MRA22/2337191). The questionnaire was based on a previously validated European survey on cariology and operative dentistry teaching conducted by the ORCA and the European Federation of Conservative Dentistry.^12^

Participants were invited to take part following a process of identifying the key member of academic teaching staff with most responsibility for teaching ETW at all UK undergraduate dental schools. Participants were recruited by email contact with informed consent and invited to participate until all UK dental schools were represented.

All survey data were collected in the first quarter of 2024 and the results analysed anonymously which meant that no individual schools' answers were identified. The questionnaire was designed, piloted and amended by all authors. The questionnaire was administered online by Stockdale Martin, a dental healthcare company. The questionnaire investigated didactic and clinical teaching of ETW of undergraduate dental learners and included both qualitative and quantitative data.

The questionnaire covered five sections which were designed to explore participants' opinions, attitude and experience of teaching ETW. The questions were structured using Likert scale responses with one free-text question at the end. Section I collected the name of the UK dental school represented. Section II included questions relating to the participant's experience using international curricular guidance for teaching the condition. Section III explored participants' experience of teaching the condition, regarding the overall curricular experience of learners and specific questions which related to four of the domains of the CCC.^9^ These were:

- Domain I – the knowledge base
- Domain II – risk assessment and diagnosis
- Domain III – decision making and preventive therapy
- Domain – V evidence-based practice
- Domain IV on surgical decision-making was specifically excluded from the questionnaire as the restorative treatment of ETW was not within the scope of this present study.

Section IV investigated the learner's experience of didactic and clinical teaching of the condition. Finally, Section V investigated the overall curricular experience of ETW teaching in the participant's dental school. Free text responses were used to understand the dental school's priorities in this field. The full list of questions is available using this link: https://www.surveymonkey.com/r/C7K8C6Q. Where appropriate, statistical analyses were performed for the quantitative data by allocating scores^1^^,^^2^^,^^3^^,^^4^^,^^5^ to the Likert scale responses and as the responses were non-parametric, a Kruskal-Wallis test with Dunn's correction applied for multiple comparisons using statistical software (GraphPad Prism 10 Windows, GraphPad Software, Boston, Massachusetts USA, www.graphpad.com).

## Results

The key results from the survey were as follows. A response was received from all 16 UK undergraduate dental schools, thus 100% of the key academic leads for teaching and curriculum design for ETW were represented. Fifty-three percent of UK dental schools were very/familiar with international educational guidance for ETW teaching and 57% agreed/strongly agreed that they followed a dedicated ETW teaching structure. Use of the CCC was widespread (77% any use). Teaching terminology varied across the UK; while 46% of schools preferred teaching the term ‘tooth wear' only 15% favoured the term ‘erosive tooth wear'. Sixty-nine percent of schools were always teaching the concepts of erosion, abrasion and attrition whereas abfraction was taught statistically less often (*p* <0.05). The coverage of aetiology was consistently strong for diet and reflux (‘always' taught in 92% of schools), bruxism and tooth brushing (‘always' taught in 85% of schools), which were statistically significantly greater than teaching of genetics and acquired enamel pellicle (*p* <0.001). The expectations for ETW screening and risk-assessment expectations were universal (100% expected students to be familiar/very familiar with screening and risk-assessment). Ninety-two percent of teachers always/often taught the BEWE Screening tool. Finally, respondents were statistically significantly more likely to expect their students to be using the BEWE (75% very likely/likely to be used clinically) in contrast to no formal screening being undertaken (100% unlikely/very unlikely to be used clinically) (*p* <0.001).

### Section II results: using international curricula guidance for teaching erosive tooth wear

**Fig. 3:**
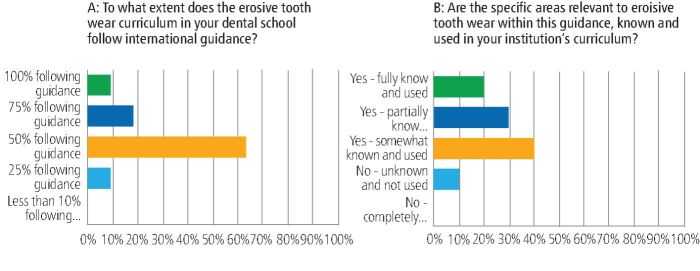
Responses to the questions pertaining to the UK dental school experience of teaching using the 2010 core cariology curriculum

Fifty-seven percent of respondents agreed/strongly agreed that their ETW teaching followed a dedicated subject specific structure (similar to periodontology or cariology). However, there was a significant proportion who felt otherwise: 29% disagreed and 14% could neither agree nor disagree. Similarly, while 53% of UK dental schools reported high levels of familiarity with international curricula guidance for teaching ETW, no dental school reported being completely unfamiliar. Of those who were using some form of international curricula guidance, almost 77% responded that they were using the CCC guidance,^9^ whereas only 8% were completely unaware of this specific guidance document Table 1. Ninety-one percent of UK dental schools reported using the guidance for 50% or more of their teaching (Fig. 3A), which was further supported by the finding that 90% reported using the specific recommendations on ETW in the CCC guidance. In contrast, only 10% claimed not to use this ETW guidance at all (Fig. 3B).

**Table 1:** Responses to the question ‘is the core cariology curriculum known and used in your institution's curriculum?'^2^^,^^3^

**Is the core cariology curriculum known and used in your institution's curriculum?**	**% response (n = 13)**
Yes – fully known and used	23.08
Yes – partially known and used	23.08
Yes – somewhat known and used	30.77
No – unknown and not used	15.38
No – completely unknown and not used	7.69

When participants were surveyed regarding their preferred terminology for teaching the condition, 46% preferred the term *‘*tooth wear' whereas 23% preferred ‘tooth surface loss', 15% *‘*erosion/attrition/abrasion' and 15% ‘erosive tooth wear'.

### Section III results: teaching the knowledge base of erosive tooth wear

**Fig. 4:**
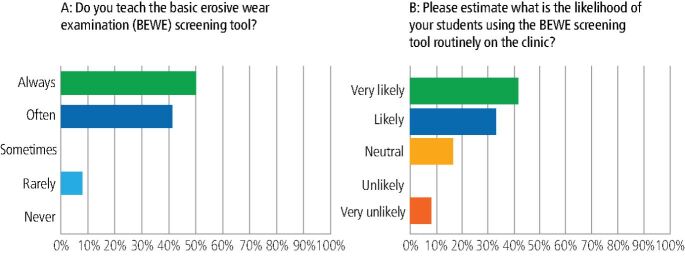
Responses to the questions pertaining to the UK dental school experience of teaching and using the basic erosive wear examination for screening of erosive tooth wear

When asked about teaching the knowledge base both didactically and clinically, 69% of schools were always teaching the concepts of erosion, abrasion and attrition whereas abfraction was taught statistically less frequently (53% sometimes/rarely taught) (*p* <0.05). As shown in Table 2, 92% of dental schools revealed that they were always teaching diet and reflux and 85% were always teaching bruxism and toothbrushing as aetiological/causative factors whereas in contrast teaching of genetics and biofilm/acquired enamel pellicle was 69% rarely/never taught (*p* <0.001). One hundred percent considered the clinical appearance of ETW as being very important when carrying out a risk assessment and diagnosis. One hundred percent of respondents expected their learners to be very familiar/familiar with risk assessment of ETW with 92% always/often teaching the BEWE screening tool, whereas in contrast only 8% rarely taught the BEWE screening tool (Fig. 4).

**Table 2:** Responses to the question ‘within your curriculum and teaching, how frequently are the following aetiology/causative factors of the condition taught?'

	**% Always taught (n)**	**% Often taught (n)**	**% Sometimes taught (n)**	**% Rarely taught (n)**	**% Never taught (n)**
Diet^ab^	92.31 (12)	7.69 (1)	0 (0)	0 (0)	0 (0)
Gastric reflux^cd^	92.31 (12)	0 (0)	7.69 (1)	0 (0)	0 (0)
Bruxism^ef^	84.62 (11)	15.38 (2)	0 (0)	0 (0)	0 (0)
Toothbrushing^gh^	84.62 (11)	15.38 (2)	0 (0)	0 (0)	0 (0)
Xerostomia	46.15 (6)	15.38 (2)	30.77 (4)	7.69 (1)	0.00
Genetics^aceg^	0 (0)	7.69 (1)	23.08 (3)	61.54 (8)	7.69 (1)
Acquired enamel pellicle/biofilm^bdfh^	15.38 (2)	7.69 (1)	7.69 (1)	53.85 (7)	15.38 (2)
Matching superscript letters within leftmost column indicate statistically significant differences between responses (*p* <0.01)

When teaching clinical decision-making for ETW, 100% of respondents reported that they considered diagnosis to be very important/important for clinical decision-making. When asked about the use of the evidence base for teaching ETW, only 50% of educators felt that their learners would be likely to be aware that ETW is the third most observed condition observed in the UK. Seventeen percent felt that their students were always following evidence-based clinical practice when managing ETW.

### Section IV results: didactic and clinical teaching of erosive tooth wear

When the timing of didactic ETW teaching in the British dental school's curriculum was surveyed, there was a distribution of responses across years and schools, without a clear picture emerging. However, 67% of the dental schools reported having preclinical hands-on workshops focused on ETW. When specifically asked about clinical expectations of their students (Table 3), 75% felt that their dental students were very likely/likely to be using the BEWE routinely on the clinic which was statistically greater than those who expected no formal screening to be undertaken (100% unlikely/very unlikely) (*p* <0.001). Similarly, 73% reported their learners would be very likely/likely to use clinical estimates of severity on the clinic which was statistically greater than those who expected no formal screening to be undertaken (*p* <0.001).

**Table 3:** Responses to the question ‘please estimate what is the likelihood of your learners using the following screening tools routinely on the clinic?'

**Responses**	**% Very likely (n)**	**% Likely (n)**	**% Neutral (n)**	**% Unlikely (n)**	**% Very unlikely (n)**
Basic erosive wear examination^a^	41.67 (5)	33.33 (4)	16.67 (2)	0.00	8.33 (1)
Clinical estimates of severity (e.g. mild/moderate/severe, low/high, active/inactive)^b^	36.36 (4)	36.36 (4)	18.18 (2)	0.00	9.09 (1)
Smith And Knight tooth wear index	18.18 (2)	0.00	18.18 (2)	27.27 (3)	36.36 (4)
No formal screening undertaken^ab^	0.00	0.00	0.00	36.36 (4)	63.64 (7)
Matching superscript letters within leftmost column indicate statistically significant differences between responses (*p* <0.001)					

In terms of preventive therapy for ETW, 80% responded that their students would always/often recommend a high fluoride toothpaste, 62% a regular fluoride toothpaste, 30% a regular fluoride mouthrinse and only 18% a high fluoride mouthrinse. When asked about which professionally applied fluoride delivery system students routinely advise, 83% felt that their students would always or often recommend a fluoride varnish whereas only 33% felt that their students would recommend a remineralising agent such as casein phosphopeptide-amorphous calcium phosphate.

### Section V results: overall experience of teaching of erosive tooth wear

Ninety-two percent felt it was very important/important for UK dental schools to develop and maintain academic and clinical expertise in ETW. When asked for further comments regarding how ETW teaching is implemented (didactically and clinically) at UK dental schools (e.g., successes, failures, proposed improvements, barriers encountered, etc), a range of free text responses were received as shown in Table 4. A key finding was that ‘I do not feel the cariology curriculum covers tooth wear in enough detail'.

**Table 4:** Responses to the question ‘do you have any further comments regarding how erosive tooth wear teaching is implemented (didactically and clinically) at UK dental schools (e.g., successes, failures, proposed improvements, barriers encountered, etc.)

‘Tooth wear is frequently multifactorial in origin even when primarily erosive, whole patient approach required and Interprofessional Education approach applied. Patients' scenarios (should) include sleep apnoea, obesity etc'
‘The terminology used could be updated. The learners found it helpful to see different erosive tooth wear clinical patient cases and their management with photographs in their *Treatment of Worn Teeth Handbook*'
‘Improvements have been proposed and some introduced since last academic year'
‘I do not feel the cariology curriculum covers tooth wear in enough detail'
‘I think it needs an update especially on the clinical management aspect'

## Discussion

The aim of this cross-sectional survey was to explore the experience of key academic educators teaching and designing educational curricula in the field of ETW in UK undergraduate dental schools. A key focus was to understand levels of awareness and adherence to international educational guidance, specifically the CCC developed by the ORCA and the Association of Dental Education in Europe and ETW screening tools, namely the BEWE. Thus, the main findings were that firstly that international guidance on ETW education is changing UK teaching practice and secondly that one screening tool to capture ETW, the BEWE, is used almost universally across the UK for teaching.

The findings of this study add important information into what is being taught in ETW and how it is being taught. Some of the challenges and the limitations of the current educational landscape for ETW were identified especially regarding adoption and usage of terminology. It is important to note that recent consensus terminology guidance^2^ defines tooth wear as ‘the cumulative surface loss of mineralised tooth substance due to physical or chemo-physical processes (dental erosion, attrition, abrasion)'. In contrast, ETW is defined as ‘tooth wear with dental erosion as the primary etiological factor'. Thus, it is perhaps unsurprising that when participants were surveyed regarding their preferred terminology for teaching the condition, 46% preferred the term ‘tooth wear' whereas only 15% ‘erosive tooth wear'. While this can be explained due to the former term being open as to the cause of the condition, it is important to note that prevalence data support the role of acid erosion being the predominant aetiological factor in tooth wear.^2^ However, while only 15% of respondents reported using ETW in their teaching, 92% of respondents were teaching that dental erosion from exposure to acids in the diet or from the stomach is the primary causative factor of ETW. Therefore, even if UK teaching currently does not have consensus around the terminology there is an emerging picture of the importance of teaching control of dietary acids as the key preventive message surrounding the condition, which is aligned with the evidence base.

In terms of challenges to uptake of the international educational curricula, the finding that 53% of UK dental schools report high levels of familiarity with international guidance for teaching ETW is reflected by worldwide global experiences with implementing research-informed educational developments.^11^ It is encouraging that in this study, 91% of UK dental schools who were familiar with guidance report using it for 50% or more of their teaching, which was further supported by the finding that 90% reported using the specific recommendations on ETW in the CCC guidance. This perhaps reflects the rapidly emerging evidence base surrounding ETW to support teaching this important condition.

Following the introduction of the BEWE in 2007, dental schools globally have updated their curricula to include a greater emphasis on screening and risk assessment with generally positive results. In a 2024 Brazilian study,^13^ training on use of the BEWE screening tool improved the diagnostic ability of undergraduate dental students when examining ETW.^13^ In the UK, undergraduate dental students risk assessed and screened for greater levels of tooth wear once the BEWE score was added to the electronic health care record software.^14^ A similar picture is seen in UK general dental practice with the BEWE the most commonly used screening tool, especially among recently graduated dentists^4^^,^^15^ and is recommended by specialist society guidelines on ETW management.^16^ It is important to note that the BEWE is mainly quantification (giving the assessed tooth wear a number) and some risk assessment, but not qualification (discrimination between mechanical and erosive causes), therefore determining the relative contribution of the different causes of tooth wear is not part of the design of the tool.

A key limitation of this survey was that it did not consider the views of the learners, however a range of studies have shown growing awareness of dental students^17^^,^^18^ regarding the aetiology and prevention of ETW. Another limitation is that while 100% response rate was achieved; and from all 16 UK undergraduate dental schools, it represents around 33% of all UK educators teaching in this field according to the estimate of approximately three key educators in each dental school teaching ETW and undertaking curriculum design in this field.

The present study has shown that teaching emphasised the role of diet and reflux as well as bruxism and toothbrushing as aetiological/causative factors; whereas in contrast teaching of genetics and biofilm/acquired enamel pellicle was 69% rarely/never taught (*p* <0.001). Therefore, while these data suggest that a wide range of aetiologies are considered, it is not known what extent this feed into prevention in the clinic. Is it important to consider that overwhelming evidence regarding the nature of ETW being an acid mediated phenomenon requires reiteration of the importance of clinician training that the condition is slowly progressing, irreversible but likely highly controllable with diet/lifestyle modification: that is, potentially easily stopped once the key aetiologies are controlled.^19^

Further work is needed to understand how the findings are reflective of what is being taught in UK and worldwide dental schools overall, and ongoing scoping surveys and educational research is being conducted to determine the best way to structure an ETW curriculum, specifically surrounding the nature of both hands-on and didactic teaching. While 67% of respondents in this present survey reported undertaking hands-on workshops in ETW before going onto clinic, it was not clear what these focused on. There is a need to develop educational resources that can be provided in a range of languages, which is work that is ongoing via educational bodies such as the ETW Foundation among other key stakeholders and societies.^20^

## Conclusion

This survey has found high levels of engagement among UK dental schools with regards to teaching ETW and that the condition is being taught in a sophisticated fashion in line with the latest evidence base. Ninety-two percent of respondents are using the BEWE tool for teaching and 75% reporting that their learners are using the BEWE routinely on the clinic. However, further work is needed to understand what hands-on teaching is happening and why there are variable degrees of confidence in the teaching of the condition. How the use of terminology should evolve is an active ongoing debate, with international groups (namely the ORCA and The Association for Dental Education in Europe) continuing to update their guidance to enhance the specific guidance on teaching ETW. This will help educators keep abreast of developments to ensure their learners are provided with the most up-to-date research-informed education in the field of ETW.

## Data Availability

The primary data can be shared on request with the participant information anonymised.
